# Frequency Diverse Array and Spotlight Synthetic Aperture Radar 2D Imaging Based on Multiple Repeated Subpulses

**DOI:** 10.3390/s25041075

**Published:** 2025-02-11

**Authors:** Qinlin Li, Kefei Liao, Ningbo Xie, Hanbo Chen

**Affiliations:** 1 School of Information and Communication, Guilin University of Electronic Technology, Guilin 541004, China; liqinlin@guet.edu.cn (Q.L.); xieningbo@guet.edu.cn (N.X.); chenhanbo@guet.edu.cn (H.C.); 2 Guangxi Intelligent Electromagnetic Spectrum Perception and Control Technology Engineering Research Center, Guilin 541004, China

**Keywords:** frequency diverse array, higher energy accumulation, BP algorithm, spotlight synthetic aperture radar, deconvolution algorithm

## Abstract

Frequency diverse array (FDA) beams show an “S” shape in space and cannot form a spot beam; thus, they cannot be directly combined with spotlight synthetic aperture radar (SSAR). In this paper, we propose a 2D imaging system emitting multiple repeated subpulses using an FDA and spotlight synthetic aperture radar (MRS-FDA-SSAR). This system carries the FDA on an airborne platform and uses the frequency difference between the array elements to synthesize broadband signals and obtain the distance-direction resolution, and then it uses a synthetic aperture technique to obtain the azimuth-direction resolution. Subsequently, 2D imaging results are obtained using the BP algorithm. A deconvolution algorithm is introduced to address the problem of high target sidelobes in the BP imaging results, which can result in the masking of weak targets. This allows 2D imaging results to be obtained with lower sidelobes. Finally, the MRS-FDA-SSAR model was simulated in experiments to verify its effectiveness.

## 1. Introduction

Synthetic aperture radar (SAR) technology, which uses virtual array elements to synthesize a large aperture, plays a crucial role in radar imaging [1,2]. Spotlight SAR can be used to obtain a wide directional beam by controlling the direction of the beam, which can increase the irradiation width of the antenna beam in the imaging area in a short time; thus, a short antenna can obtain a higher resolution. This is an important aspect of synthetic aperture radar [3,4]. The traditional spotlight SAR system uses a phased array to obtain a high range resolution via the transmitted broadband signal [5]. This not only requires expensive hardware but also presents the problem of inflexible beam direction adjustments. Technical difficulties need to be overcome in the development of SAR in order to flexibly set the transmitted beam direction to form a continuous coverage area, reduce the system’s power consumption, and obtain full-range, high-resolution 2D imaging results [6].

Researchers have previously studied the combination of phase control, MIMO, FDAs, and SAR [7,8]. Frequency diverse arrays (FDAs) are based on the idea of “space for bandwidth”, in which narrowband signals emitted from different spaces and narrowband signals with multiple carrier frequencies are combined into wideband signals [9]. The launch of a narrowband signal reduces the requirements for the transmitter. Applying FDAs to SAR imaging allows the radar to transmit a single narrowband signal at a single carrier frequency for each array element and synthesize the broadband signal from different carrier frequency echoes transmitted from different array elements. By introducing the array, a two-dimensional resolution of the target is possible. The relative motion between the target and the array forms a synthetic aperture, ultimately achieving the capability to image the target.

FDAs have been applied in the study of SAR imaging; most studies use SAR in the band mode to solve the problem of blurred distances [10]. There have been fewer studies on FDA applications using spotlight SAR models. The imaging resolution of spotlight SAR for the two-dimensional imaging of interested areas is superior. Thus, an imaging model formed by the combination of FDAs and spotlight SAR has a higher application value.

Although FDAs can avoid the high cost of hardware and are characterized by flexible bandwidth settings, the beam of an FDA does not have stable directivity and cannot be directly applied to the spotlight SAR model [11]. For this reason, this paper introduces an FDA that can transmit multiple repeated subpulses into the 2D imaging system of spotlight SAR, forming a 2D imaging system based on MRS-FDA-SSAR. This system carries the MRS-FDA on a carrier platform; the FDA adopts multiple repeated subpulses, and the movement of the carrier platform ensures that the beam is always aligned with the center of the imaging area. The transmission of multiple repeated subpulses effectively avoids the problem of the high costs caused by the array element transmitting broadband signals, and the system can achieve the 2D imaging of a ground area through the flexible control of the spotlight orientation with imaging algorithms [12].

This paper is arranged as follows: In Section 2, the MRS-FDA-SSAR-based signal model is presented. Section 3 introduces two imaging algorithms, the BP algorithm and the deconvolution algorithm, which are applied to the above model, along with a detailed analysis of the imaging resolution. In Section 4, an experimental simulation of the two algorithms used for MRS-FDA-SSAR 2D imaging is described; this section then compares the imaging results of the two algorithms and demonstrates that the deconvolution algorithm can achieve lower sidelobes to obtain better imaging results. A summary of this work is provided in Section 5.

## 2. Preliminaries

### 2.1. Imaging Model

**Figure 1:**
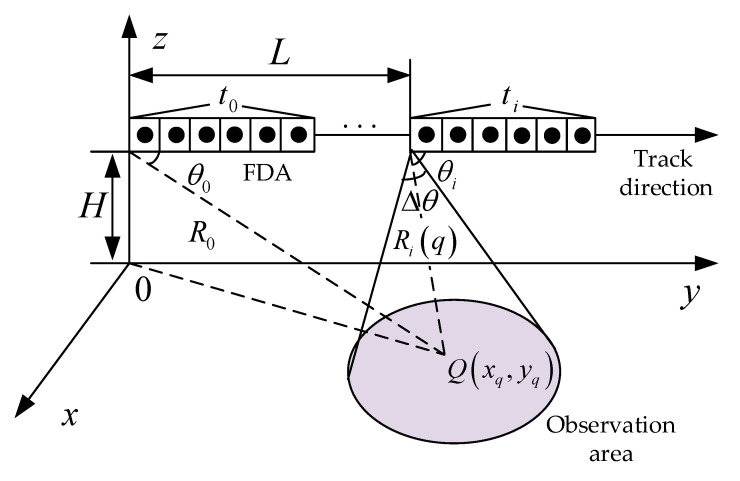
Imaging model of MRS-FDA-SSAR.

Spotlight SAR is an imaging mode that can obtain a high resolution in the angle dimension. A characteristic of this imaging mode is that the radar beam should always point at the imaging area during the imaging time [13]. Traditional spotlight SAR uses a phased array to transmit a wideband signal and obtain a directional beam, necessitating significant hardware requirements and a complex interpolation algorithm to increase the computational complexity [14]. In this study, an MRS-FDA was introduced to form a directional focusing beam and complete the construction of an MRS-FDA-SSAR imaging model. This model is shown in Figure 1. In this model, the range resolution is obtained using the property of the FDA’s space bandwidth exchange. The bandwidth size and the direction of the beam can be flexibly changed by controlling the parameters of the array elements. The radar is equipped with FDAs with a real aperture along the track, and a high-resolution ability in the azimuth direction is obtained using synthetic aperture technology.

In the MRS-FDA-SSAR imaging model, FDAs with uniform linear arrays are used. The frequency increment between adjacent elements is Δf, the coordinates of the center position of the array elements are (0,N/2d,H), and the target point $Q(x_{q},y_{q})$ is the center point illuminated by the radar beam. The airborne radar flies in a straight line with a uniform speed above the Y-axis; the flight speed is v; the beam always points to the center point of the imaging scene Q, which is unchanged; the length of the synthetic aperture of the radar is L; the height *H* of the radar from the ground remains unchanged; and the distance between the projection of the first array element on the Y-axis and the center of the scene is denoted by $R_{0}$. The azimuth angle of the FDA at different observation times $t_{i}$ is $\theta_{i}$, and the line-of-sight angle range of the FDA is Δθ. The N array elements are linearly arranged according to the equal distance d along the direction of aircraft motion, and the carrier frequency of the *n*-th array element is as follows:

(1) $$f_{n}=f_{0}+n\Delta f\;\;\;\;\;\;n=0,1,\cdots ,N-1$$

The single frequency signal emitted by the *n*-th array is as follows:

(2) $$X_{n}(t_{i})=\exp\left(j2\pi f_{n}t_{i}\right)\;\;\;i=1,2\ldots ,I$$

The distance from target point Q to the first element after $t_{i}$ time $R_{i}\left(q\right)$ can be expressed as follows:

(3) $$R_{i}\left(q\right)=\sqrt{x_{q}^{2}+H^{2}+{\left(y_{q}-vt_{i}\right)}^{2}}$$

The length of the target distance from the *n*-th array element after $t_{i}$ moments is expressed as follows:

(4) $$R_{i,n}(q)=\sqrt{x_{q}^{2}+H^{2}+{\left((y_{q}-nd\sin\theta_{i})-vt_{i}\right)}^{2}}$$

**Figure 2:**
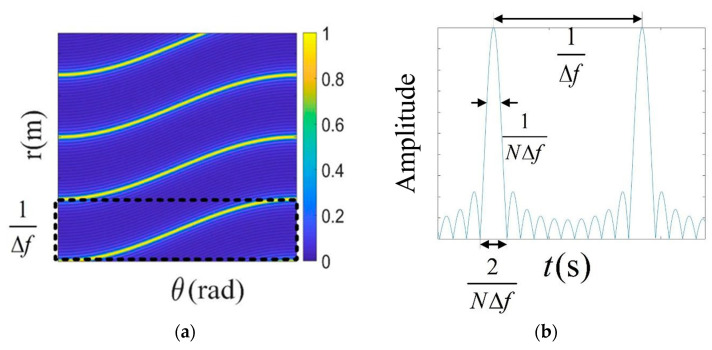
FDA beam temporal and periodicity analysis: (**a**) FDA beam pattern; (**b**) FDA beam temporal periodic analysis.

For a static goal in space, the FDA beam maintains an unchanged distance and angle; the minimum cycle is found to be 1/Δf. By intercepting the beam chart of the cycle time, the pattern displayed in Figure 2a is formed. In Figure 2b, the beam during a time cycle only shows only one mainlobe at the same angle. In other time cycles, the beam forms the mainlobe at other angles. It is difficult to complete the continuous exposure of the target.

**Figure 3:**
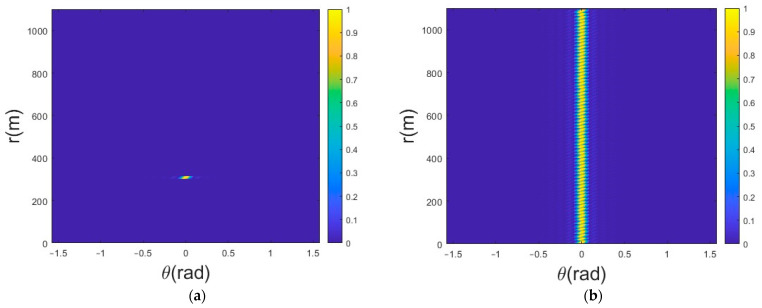
Multiple repeated subpulse beam patterns: (**a**) beam pattern of a single effective subpulse; (**b**) beam pattern of repeating effective subpulses.

For far-field targets, the mainlobe period of an emitted beam is regarded as the effective period; the mainlobe is intercepted and repeated over a period of time, and the beam superposition forms a columnar directional beam. Only one mainlobe in the intercepted beam is intercepted for transmission. An intercepted single mainlobe is shown in Figure 3a. The beam can pass through a target point to cause the phase change in the echo signal at the target position. This enables the recording of the phase information of all echo signals as well as the discovery of the phase information corresponding with the target point. The mainlobe beam under the phase is then continuously repeated, finally forming a strip-like directional beam (as shown in Figure 3b). This can be called a persistent beam because of its formation characteristics; its focusing effect is similar to that of a phased array beam. Although the overall beam-gathering effect of the two beams in space is similar, the signal within the pulse of the phased array beam does not change in the azimuth in any way and is a true strip beam. An FDA emitting a persistent beam is time- and distance-dependent over a period of time and continues to exhibit variability in relation to time. The degree of the beam time variation can be reduced over the entire time period because the beam is intercepted for only a short time.

As shown in Figure 3b, the width of the mainlobe is $T_{r}=2/N\Delta f$, which is the width of the mainlobe that requires interception. According to the angle corresponding with the central target at different times, the holding beam can always point to the imaging region and the mainlobe beam at that time can be intercepted. The effective beam time range to be intercepted is as follows:

(5) $$T_{e}=[\frac{k}{\Delta f}+\frac{R_{n}(t_{i})}{c}-\frac{f_{0}d\sin\theta_{i}}{c\Delta f}-\frac{1}{N\Delta f}:\frac{k}{\Delta f}+\frac{R_{n}(t_{i})}{c}-\frac{f_{0}d\sin\theta_{i}}{c\Delta f}+\frac{1}{N\Delta f}]$$

where *k* is an arbitrary integer. Using Equation (5) to intercept the mainlobe signal in the effective period, the focused beam at different angles can be obtained by adjusting the initial phase. At different times $t_{i}$, the phase only related to the angle $\theta_{i}$ of the imaging center point can be calculated as follows:

(6) $$\Phi_{i}=2\pi (\frac{k}{\Delta f}-\frac{f_{0}d\sin\theta_{i}}{c\Delta f})$$

Finally, the effective time range $T_{e}$ is taken as the width of the focused beam. The signal is repeatedly transmitted to form multiple repeated subpulses with different initial phases. After time $t_{i}$, the *n*-th array element receives signals from different angles transmitted by targets at different positions. The obtained echo signal Y(n) can then be expressed as follows:

(7) $$Y(n)=\sum_{n=0}^{N-1}\sum_{i=1}^{I}\sum_{k=i}^{K}A_{i,n}(k)\exp\left\{j2\pi f_{n}\left(t_{i}-\tau_{n,k}\right)\right\}\exp\left(j\Phi_{i}\right)$$

(8) $$A_{i,n}\left(k\right)=\sigma_{n}\left(k\right)rect\left((t-\tau_{i,n}\left(k\right))/T_{p}\right)$$

(9) $$\tau_{i,n}\left(k\right)=2R_{i,n}\left(k\right)/c$$

where k is the total number of target points, $\sigma_{n}\left(k\right)$ is the scattering coefficient of different target points, and $\tau_{i,n}\left(k\right)$ is the size of the round-trip delay between the *k*-th target point and the *n*-th array element in the *i*-th observation. $R_{i,n}\left(k\right)$ is then the one-way distance from the *k*-th target to the *n*-th array element when the *i*-th observation is obtained. After the down-conversion ($C=e^{-j2\pi f_{0}t}$) of the echo signal Y(n), the processed echo signal becomes

(10) $$\tilde{Y}(n)=C\sum_{n=0}^{N-1}\sum_{i=1}^{I}\sum_{k=i}^{K}A_{i,n}(k)\exp\left\{j2\pi f_{n}\left(t_{i}-\tau_{n,k}\right)\right\}\exp\left(j\Phi_{i}\right)$$

The position of the target with respect to the airborne radar constantly changes, resulting in different initial phases of the target at different observation times. The initial phase of the echo signal requires compensation to facilitate the subsequent image processing of the echo signal. The echo signal output by the receiver after passing through the matched filter in the single frequency receiving mode is rewritten as follows:

(11) $$Y^{\prime }(n)=C\sum_{n=0}^{N-1}\sum_{i=1}^{I}\sum_{k=i}^{K}A_{i,n}(k)\exp\left\{j2\pi f_{n}\left(t_{i}-\tau_{n,k}\right)\right\}$$

Finally, $Y^{\prime }(n)$ is sent to the imaging processor for processing.

### 2.2. Imaging Resolution Analysis

In traditional SAR, the signal processor plays a crucial role because the transmitted signal pulse width is very large; thus, pulse compression technology is required to obtain a good resolution [15]. The narrowband signals transmitted by different elements of MRS-FDA can be obtained after frequency diverse processing, and then the range resolution can be obtained as follows:

(12) $$\rho_{r}=\frac{c}{2B}=\frac{c}{2N\Delta f}$$

**Figure 4:**
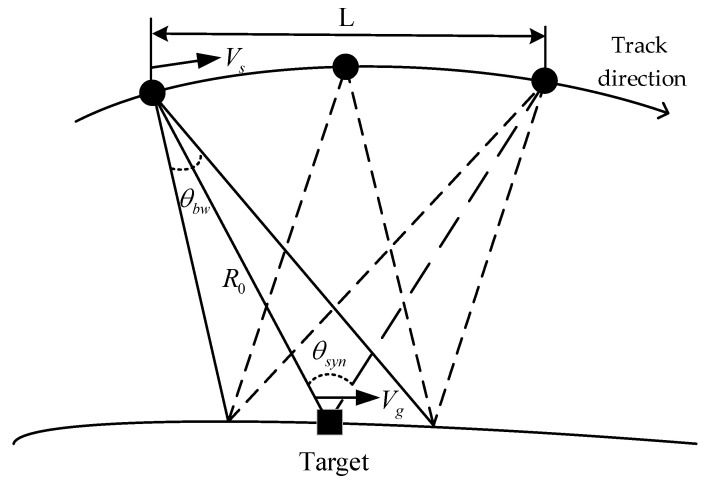
Schematic diagram of the antenna beamwidth and synthesis angle.

At different observation times, a radar beam has different Doppler shifts for a target echo signal at different azimuths. An effective azimuth resolution can be obtained using the Doppler shift to distinguish different azimuths. The azimuth resolution can be deduced from the definition of a synthetic aperture according to the width of the synthesized narrow beam. The concept of a synthetic aperture is used to synthesize large apertures to improve the resolution accordingly. The azimuth resolution can be expressed in terms of the resultant angle, which is the change in the angle of view during the time the target is illuminated by the beam. Figure 4 shows a schematic diagram of the angle, where the moving speed of the aircraft is $V_{s}$, the moving speed of the beam covering the ground is $V_{g}$, and the resultant angle $\theta_{syn}$ is as follows:

(13) $$\theta_{syn}=\frac{V_{s}}{V_{g}}\theta_{bw}$$

For the airborne spotlight SAR discussed in this paper, $V_{s}$ is approximately the same as $V_{g}$, and the resultant angle $\theta_{syn}$ is equivalent to $\theta_{bw}$. The Doppler bandwidth generated by the spotlight SAR system transmitting multiple repeated subpulses from the FDAs can be expressed as follows:

(14) $$f_{i}=\frac{k}{\Delta f}-\frac{f_{0}d\sin\theta_{bw}}{c\Delta f}$$

In the spotlight SAR model, the length of the synthetic aperture within the synthetic aperture time $T_{i}$ is L, and the line-of-sight corner range of the FDA radar is $\Delta \theta =\theta_{bw}$. The movement distance of the array after $T_{i}$ time is consistent with the distance of the synthetic aperture, which is L. When $L<<R_{i}\left(q\right)$, $T_{i}$ is approximately expressed as follows:

(15) $$T_{i}=\frac{L}{v}\approx \frac{2\sin(\frac{\Delta \theta }{2})R_{i}(q)}{v\sin\theta_{0}}$$

The azimuth resolution can then be expressed as follows:

(16) $$\rho_{a}=\frac{\lambda }{4\sin(\frac{\Delta \theta }{2})}$$

The azimuth resolution of spotlight SAR is only related to the radar’s working wavelength and the viewing angle range. Theoretically, the beam of an FDA can be scanned at all angles [16], so a continuous beam at all angle domains can also be realized by the interception of the mainlobe angle of multiple repeated subpulses of the FDA. Thus, $\Delta \theta =\theta_{bw}$ in the denominator of Equation (16) is close to the theoretically achievable maximum value of 1; finally, a high-resolution imaging result in that direction can be obtained.

## 3. MRS-FDA-SSAR 2D Imaging Method

In a traditional spotlight SAR system with a phased array, the range migration increases when the scanning angle is too large. Multiple repeated subpulses in FDAs can effectively avoid the range migration problem caused by the direct transmission of broadband signals because the beam retains the range–angle correlation property of linear FDAs in the intercepted beam. Subsequently, the combination of multiple repeated subpulses in FDAs with spotlight SAR should be considered. The advantage of a BP algorithm is that it can flexibly change the accumulation interval in the azimuth direction, which makes it more convenient to adjust the processing bandwidth in the azimuth direction [17]. It is also more suitable for acquiring the pattern of the time-varying frequency of multiple repeated subpulses from the FDAs in the azimuth direction. Therefore, the BP algorithm can be used to complete the imaging work of the MRS-FDA-SSAR system. Spotlight SAR can result in the problem of target blurring in imaging when the synthetic aperture time is too long. The multiple repeated subpulses introduced in this study are formed by capturing the mainlobe, which can prevent the problem of sidelobe superpositioning to a certain extent. At the same time, starting from the mechanism of blur generation, a deconvolution imaging algorithm is used to complete the establishment of the image-domain deblurring mathematical model. Consequently, the FDA spotlight SAR model based on multiple repeated subpulses has no blurring and presents low-sidelobe-quality imaging.

### 3.1. BP Imaging Algorithm

The space–time–frequency coupling characteristics of FDA-transmitted signals result in challenges to dimensionality reduction processing in imaging, and traditional RD and other fast dimensionality reduction algorithms cease to be applicable.

The BP algorithm is a data processing method in the time domain; there is no longer a requirement to transform the data into the frequency domain. Data can be divided according to the imaging area of the grid to obtain an accurate slant distance at each moment of the target. The slant distance can be superimposed after the processing of the echo signal for imaging.

**Figure 5:**
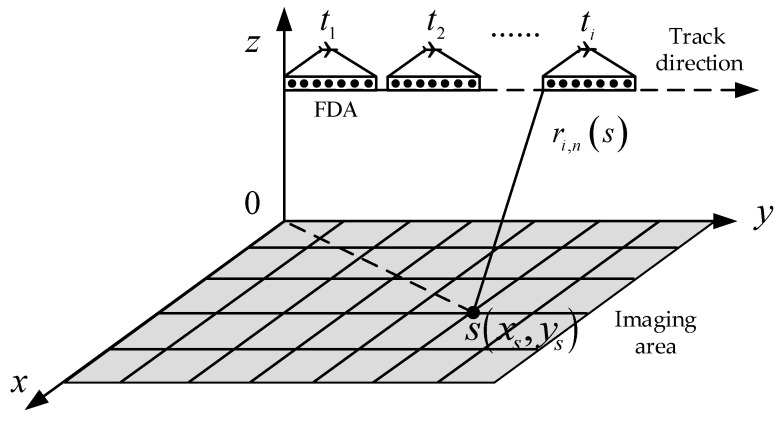
Schematic diagram of the grid division.

The entire imaging region can be divided into grids, as shown in Figure 5. In the imaging scene of the *i*-th observation time, the length of the *s*-th grid from the *n*-th element is expressed as follows:

(17) $$r_{i,n}(s)=\sqrt{x_{s}^{2}+H^{2}+{\left((y_{s}-nd\sin\theta_{i})-vt_{i}\right)}^{2}}$$

In Equation (17), $\left(x_{s},y_{s}\right)$ is the grid coordinate of *s*. The round-trip delay at this point can be expressed as follows:

(18) $$\tau_{i,n}\left(s\right)=\frac{2r_{i,n}\left(s\right)}{c}$$

(19) $$ℑ=e^{j2\pi f_{n}\frac{2r_{i,n}(s)}{c}}$$

In Equation (19), ℑ represents the phase compensation term at the *s*-th grid node. Similarly, the corresponding phases of the grid nodes at different positions and different times can be compensated for. The complex number set of each imaging grid point at a corresponding time ($t_{i}$) is then as follows:

(20) $${ℑ}_{t_{i}}=\left\{\begin{array}{l} e^{j2\pi f_{n}\frac{2r_{1,1}\left(t_{i}\right)}{c}}\;\;e^{j2\pi f_{n}\frac{2r_{1,2}\left(t_{i}\right)}{c}}\;\;\;\;\cdots \;\;\;\;e^{j2\pi f_{n}\frac{2r_{1,n}\left(t_{i}\right)}{c}}\\ e^{j2\pi f_{n}\frac{2r_{2,1}\left(t_{i}\right)}{c}}\;\;e^{j2\pi f_{n}\frac{2r_{2,2}\left(t_{i}\right)}{c}}\;\;\;\;\cdots \;\;\;e^{j2\pi f_{n}\frac{2r_{2,n}\left(t_{i}\right)}{c}}\\ \;\;\;\;\;\;\;\;\vdots \;\;\;\;\;\;\;\;\;\;\;\;\;\;\;\;\;\;\;\;\;\;\;\;\;\vdots \;\;\;\;\;\;\;\;\;\;\;\;\ddots \;\;\;\;\;\;\;\;\;\;\;\;\;\;\vdots \;\;\;\;\;\;\;\;\;\;\\ e^{j2\pi f_{n}\frac{2r_{i,1}\left(t_{i}\right)}{c}}e^{j2\pi f_{n}\frac{2r_{i,2}\left(t_{i}\right)}{c}}\;\cdots \;e^{j2\pi f_{n}\frac{2r_{i,n}\left(t_{i}\right)}{c}} \end{array}\right\}$$

The phase compensation of the echo signal corresponding with Equation (11) is then completed. The echo signals received by each imaging unit and array element in the imaging area are all superimposed to obtain $Y^{\prime \prime }(n)$. The superimposed signal amplitude is normalized and processed modulo. The final pixel value $Y_{i,n}(n)$ can then be obtained, and 2D imaging can be completed.

(21) $$Y^{\prime \prime }(n)=CS\sum_{k=i}^{K}A_{i,n}(k)\exp\left\{j2\pi f_{n}\left(t_{i}-\tau_{i,n}\right)\right\}$$

(22) $$Y_{i,n}(n)=\left|\overset{I}{\underset{i=1}{\sum }}\overset{N-1}{\underset{n=0}{\sum }}Y^{\prime \prime }(n)\right|$$

### 3.2. Deconvolution Imaging Algorithm

In the BP imaging algorithm, the 2D image of a target is composed of the gain of the echo signal and the sidelobes caused by the point spread function in the imaging region. The sidelobe of the BP imaging algorithm is very high because of the influence of the point spread function, which is the fundamental reason why a target with a weak scattering coefficient is masked [18]. We introduced a deconvolution imaging algorithm to improve the influence of beam sidelobes on the final imaging results to obtain high-quality imaging results from the MRS-FDS-SSAR model. The basic concept of a deconvolution algorithm is to continuously eliminate the influence of the point spread function on the imaging results to obtain the maximum value and the inverse filtering of an image, using the inverse filtering signal to complete the suppression of the sidelobes in the image. The deconvolutional algorithm is suitable for the removal of the side petals from the SAR imaging results completed using the BP algorithm.

The BP imaging result is obtained by convoluting the true scattering coefficient with the point spread function (PSF) as follows:

(23) BP=σ⊗PSF

The scattering coefficients at all times can also be accumulated by multiplying the point spread function corresponding with each pixel:

(24) $$BP=\sum_{i}\sigma_{i}PSF_{i}$$

If the point spread function has unwanted secondary responses (sidelobes, feathers, etc.) because of a lack of spatial frequency components, deconvolution algorithms can be used to eliminate the effects of these secondary responses.

The deconvolution algorithm must provide a different estimate each time to improve the estimation accuracy of the scattering coefficient and reduce the error of each estimation. This ensures that better scattering coefficients can be obtained each time. Assuming that an estimate of the scattering coefficients present in the entire imaging scene has been obtained, the corresponding residual can be calculated as follows:

(25) $$C=\sum_{i}(\sigma_{i}-{\sigma^{\prime }}_{i})PSF_{i}$$

**Figure 6:**
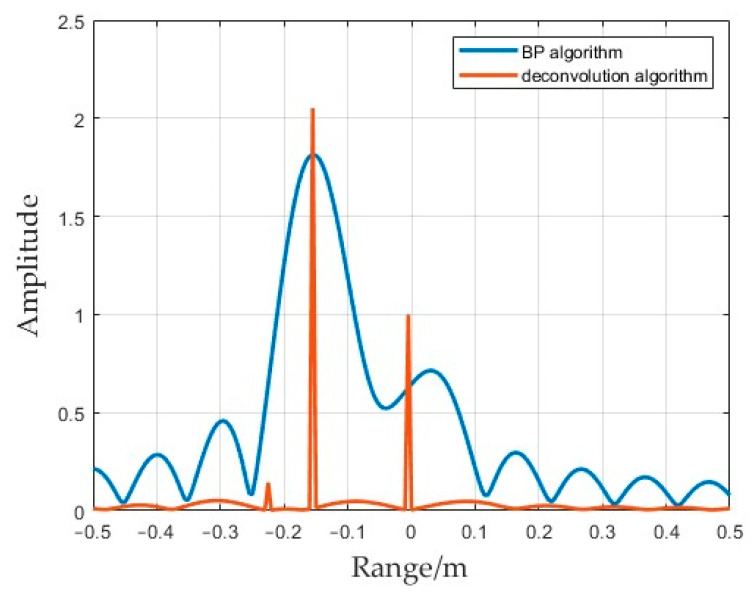
Results of range dimension slices. The blue line is the azimuth slice diagram of the BP algorithm and the red line is the azimuth slice of the deconvolution algorithm.

The size of the residual result corresponds with the error of the estimated value, and the residual is equal to zero when an exact estimate is completely reached. Figure 6 shows a slice diagram of two target points in the azimuth direction; the scattering coefficient values of the two targets are 1 and 2, respectively. The blue line in Figure 6 shows that the scattering coefficients of the target points directly imaged by the BP algorithm were affected by the sidelobes and that their respective peak values were biased. The sidelobes close to the target had a significant influence on the intensity. The red line in Figure 6 shows the azimuth slice obtained using the deconvolution imaging algorithm; the influence of sidelobes was suppressed in the imaging results, and an accurate reconstruction of the peak could be completed.

The specific implementation steps of the deconvolution imaging algorithm are described below.

Step 1: Input the target point into the FDA spot beam of an SAR model that emits multiple repeated subpulses and use the BP algorithm to image the target and calculate $Y_{i,n}(n)$. The observation matrix of the echo signal obtained by calculating the delay from the array element to the imaging grid is

(26) $$\Phi =\left[\begin{array}{cccc} e^{j2\pi f_{n}\tau_{1,1}} & e^{j2\pi f_{n}\tau_{1,2}} & \cdots & e^{j2\pi f_{n}\tau_{1,N_{t}\times N_{r}}}\\ e^{j2\pi f_{n}\tau_{2,1}} & e^{j2\pi f_{n}\tau_{2,2}} & \cdots & e^{j2\pi f_{n}\tau_{2,N_{t}\times N_{r}}}\\ \vdots & \vdots & \ddots & \vdots \\ e^{j2\pi f_{n}\tau_{N,1}} & e^{j2\pi f_{n}\tau_{N,2}} & \cdots & e^{j2\pi f_{n}\tau_{N,N_{t}\times N_{r}}} \end{array}\right]$$

In Equation (26), the time delay $\tau_{N,N_{t}\times N_{r}}$ is caused by the distance difference between the *N*-th array element and the $N_{t}\times N_{r}$ grid. The point spread function for the *k*-th objective is expressed as follows:

(27) $$PSF_{k}=\Phi \left(:,k\right)\Phi^{*}$$

where $\Phi \left(:,k\right)$ represents the *k*-th column of matrix Φ and ${\left[•\right]}^{*}$ represents the conjugate matrix.

Step 2: Take $Y_{i,n}(n)$ as the initial input matrix $D_{i}$ and the matrix when i=1 as the initial processing matrix. Continuously search for the maximum value from the initial matrix $D_{i}$ and record the position information of the maximum value; i.e., $\left(d_{\max},j\right)=\max\left(\left|D_{i}\right|\right)$ can also be expressed as $d_{\max}=D_{i}\left(k\right)$. The scattering coefficients of different targets can be estimated by dividing the maximum value of the processing matrix $d_{\max}$ by the imaging gain. In this model, the gain size caused by the influence of the BP imaging dimension is $N^{2}$; thus, the target scattering coefficient is as follows:

(28) $${\hat{\sigma }}_{k}={d_{\max}/N}^{2}=D_{i}\left(k\right)/N^{2}$$

Step 3: Multiply the PSF with the newly estimated scattering coefficients to obtain the PSF influence matrix:

(29) $$B_{k}={\hat{\sigma }}_{k}PSF_{k}={\hat{\sigma }}_{k}\Phi \left(:,k\right)\Phi^{*}$$

Step 4: Subtract the influence matrix $B_{k}$, calculated at this point with $D_{i}$, and determine whether the new processing matrix is less than a certain threshold; return to the second step if it is greater. If it is less than the threshold, add it to the newly subtracted $B_{k}$ and return to the matrix $D_{i}$ as a new processing matrix (${D_{i}}^{\prime }$) to continue the operation.

Step 5: Read the position of the maximum point recorded in the second step, re-estimate the scattering coefficient of the peak point, and complete the update of all position information.

Step 6: Continue to repeat the above process of five steps until the latest processing matrix is equal to a certain threshold. The scattering coefficients of all target points in the iterative process are written into the newly generated coefficient estimation matrix of target scattering according to their position information.

Step 7: Multiply the newly estimated scattering coefficient from the target and the corresponding gain to obtain the matrix. Add this matrix to the latest processing matrix of ${D_{i}}^{\prime }$. At this time, the obtained matrix is used for imaging. The imaging reconstruction matrix can then be expressed as follows:

(30) $$G=N^{2}{\hat{\sigma }}^{\prime }+{D_{i}}^{\prime }$$

**Figure 7:**
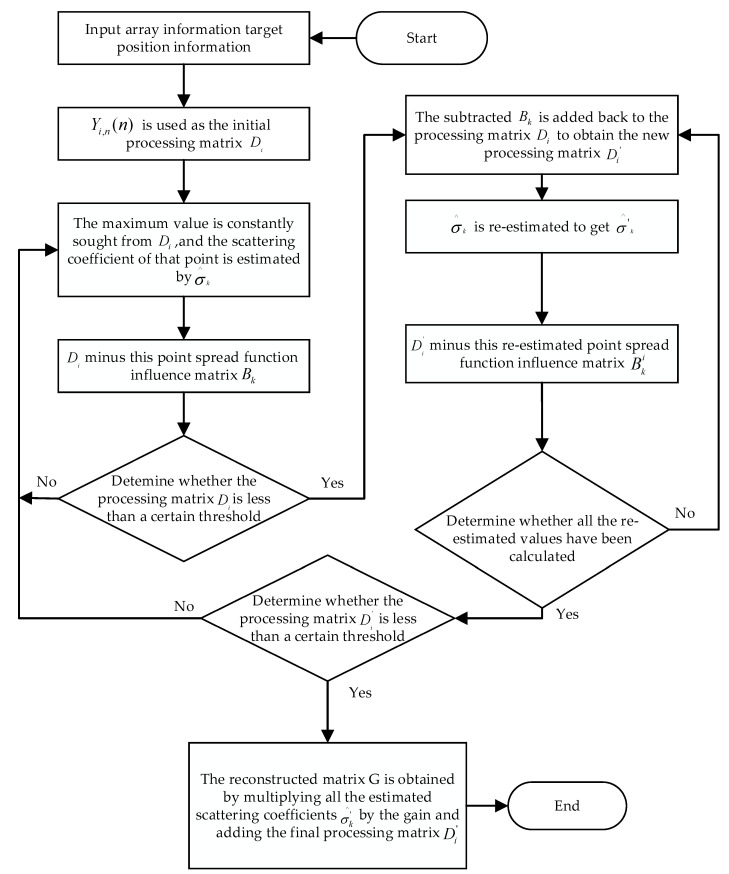
The overall workflow of the deconvolution algorithm.

The overall workflow obtained by applying the deconvolution algorithm to the MRS-FDA-SSAR imaging system is shown in Figure 7.

## 4. Simulation Experiment

A simulation experiment of the two-dimensional imaging model of MRS-FDA-SSAR was performed. The main simulation parameters of the model were set according to the values in Table 1.

**Figure 8:**
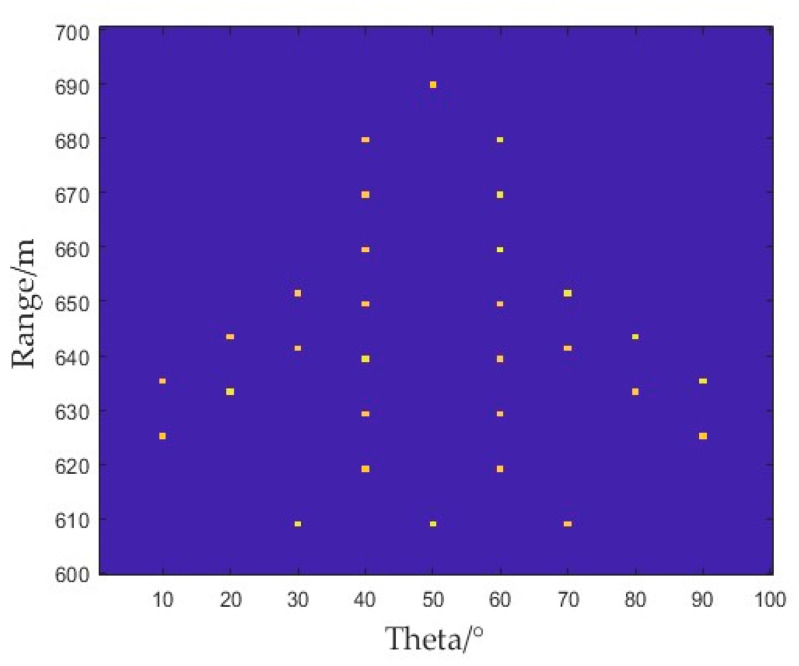
Original imaging scene.

The imaging area of the simulated imaging scene was divided into small grids of 100 × 100. The value range of the squint imaging area in the Y-axis direction was set at 600 ~ 700 m, and the range in the X-axis direction was $1\sim 100^{{}^{\circ}}$. The original scene of an aircraft scattering point model is shown in Figure 8.

**Figure 9:**
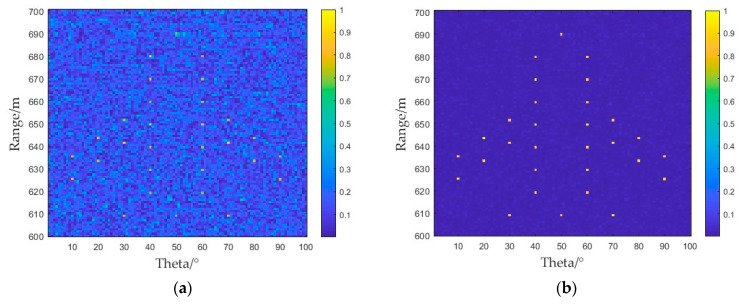
MRS-FDA-SSAR 2D imaging results: (**a**) BP algorithm imaging results; (**b**) deconvolution algorithm imaging results.

Using the MRS-FDA-SSAR radar squinted imaging model, imaging results were obtained by imaging the original scene point of Figure 8 with the BP imaging algorithm and the deconvolution imaging algorithm, as shown in Figure 9.

As shown in Figure 9a, the imaging results obtained using the BP algorithm have background blurring, which prevents the accurate imaging of the target. The long duration of the synthetic aperture leads to the superposition of peak sidelobes. When imaging complex scenes, a target with a small scattering coefficient is obscured, and high-quality imaging cannot be achieved. As shown in Figure 9b, the results obtained by the imaging method of deconvolution after BP processing were used. This imaging method continuously eliminated the influence of the image’s point spread function and extracted the corresponding peak coefficient. This avoided the problem of sidelobe superpositioning to a certain extent and effectively solved the problem of background blurring to obtain high-quality imaging results.

**Figure 10:**
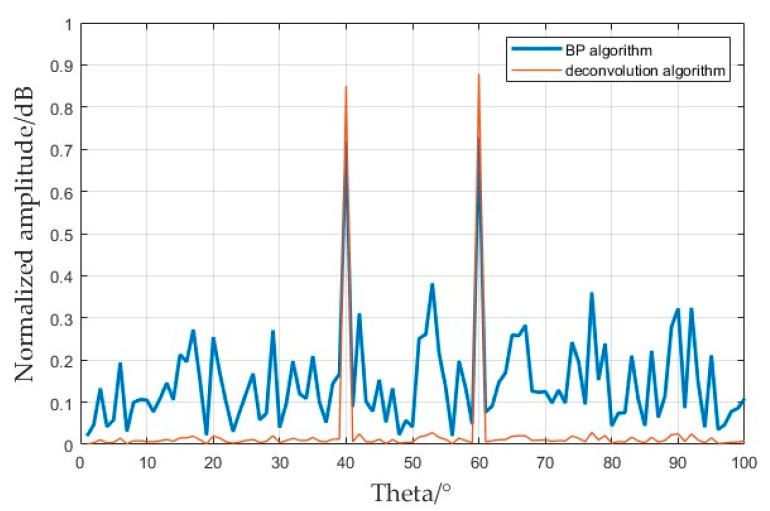
Slice diagram of the angle dimension. The blue line represents the BP algorithm imaging results, and the red line represents the deconvolution algorithm imaging results.

To specifically analyze the problem of sidelobe suppression, the imaging signal at a distance of 640 m (from Figure 9) was intercepted and compared, and a one-dimensional slice diagram was obtained, as shown in Figure 10. Two target points were set at a distance of 640 m. The mainlobe of the FDA transmitting multiple repeated subpulses was captured; the results obtained by the BP 2D imaging algorithm are shown by the blue line in Figure 10. The peak coefficient at the target point could not reach the preset value, and there were sidelobes of different sizes at other angles. The slice image obtained after processing using the deconvolution algorithm is shown by the red line in Figure 10, which shows that the target point was accurately located and basically had no sidelobes. Multiple repeated subpulses provided the retention of the mainlobe beam. After using the BP imaging algorithm, the residual sidelobes continued to lead to the superposition of sidelobes in the case of multiple targets, and the intensity of the superposition of the sidelobes was between 0.2 and 0.4. In the results obtained using the deconvolution algorithm, the intensity of the sidelobes decreased to less than 0.1, which further reflects that the convolutional 2D imaging algorithm had a better ability to eliminate sidelobes and optimize background shading.

From a comparison of the image quality assessment parameters, we introduced the following two parameters to evaluate the imaging performance of the above two algorithms.

1. Target-to-Background Ratio (TBR)

(31) $$TBR=20\mathrm{lg}\left(\frac{\underset{i\in T}{\max}\left(\left|\alpha_{i}\right|\right)}{\left(1/N_{B}\right)\sum_{i\in B}\left(\left|\alpha_{i}\right|\right)}\right)$$

In Equation (31), T is the position set of all target pixel points in the simulation result graph, B is the position set of each pixel point in the imaging region, $N_{B}$ is the total number of pixel points in the imaging region, and $\alpha_{i}$ is the pixel value of the *i*-th pixel point. The higher the TBR value of an image, the more prominent the target features in the image and the easier it is to distinguish them from the background during imaging.

2. Image Entropy (ENT)

(32) $$ENT=\sum_{J}P(j)\log P(j)$$

Image entropy (ENT) reflects the information content of an image by using the statistical characteristics of the image to calculate the average information content of the image. In Equation (32), P(j) represents the proportion of pixel points with a gray value of j in all pixel points (or the probability of pixel points with a gray value of j appearing in the imaging result map) and J is the total number of gray values in the imaging result map. The larger the ENT value of an image, the richer the gray information of the image and the greater the shading information contained; the smaller the ENT value, the clearer the imaging result, with small sidelobes.

We calculated the TBR of the above imaging results as well as the entropy of the image. The results are presented in Table 2.

The index data of the two algorithms are shown in Table 2. Under the same simulation parameters, the TBR value of the deconvolution algorithm was higher than that of the BP algorithm, whereas the ENT was lower than that of the BP algorithm. This once again confirms that the deconvolution algorithm has a greater sidelobe removal effect on the signal at the target point; thus, the characteristics in the imaging result are more obvious.

In sum, a BP imaging algorithm and a deconvolution imaging algorithm were used to perform an experimental simulation in which the target position and array coefficients were maintained, respectively. By comparing the results of the two imaging algorithms, the feasibility and effectiveness of the MRS-FDA-SSAR squinting imaging model were verified.

## 5. Conclusions

In this study, we combined an FDA transmitting multiple repeated subpulses with spotlight SAR and proposed a 2D imaging method based on MRS-FDA-SSAR, aiming to address the shortcomings of traditional spotlight SAR imaging systems, such as a complex system structure at both ends of a broadband signal receiver and transmitter, and inflexible system beam-pointing control, which leads to a discontinuous imaging area. In this imaging model, the MRS-FDA is mounted on a moving aircraft. With different observation times, the persistent focus beam of the FDA always points to the imaging area, and the array can form a long aperture array structure with the movement of the platform. This effectively improves the azimuth resolution and realizes 2D imaging results.

A time-domain BP was introduced for two-dimensional imaging. However, when using the MRS-FDA-SSAR 2D imaging method, the target was blurred because of the long duration of the synthetic aperture and the superposition of sidelobes, and a weak target point was obscured when the BP algorithm was directly applied. A deconvolution algorithm was introduced to solve the above problems. By comparing single-target and multi-target azimuth slice images in simulation experiments, we verified that the imaging results of the BP algorithm contained background blurring because of the superposition of sidelobes. The deconvolution algorithm was more effective in removing sidelobes. The simulation results show that the deconvolution algorithm can effectively suppress the sidelobes, proving the effectiveness of the MRS-FDA-SSAR 2D imaging system. A hardware structure is no longer needed to alter the irradiation angle of the spotlight beam; thus, the technical complexity is reduced, as is the corresponding cost.

## Figures and Tables

**Table 1:** Simulation parameters of MRS-FDA-SSAR.

Parameter	Parameter Value
$\mathrm{Carrier}\;\mathrm{frequency},\;f_{0}$	10 GHz
Frequency offset, Δf	3.33 MHz
Pulse repeat frequency,PRF	1000 Hz
Flight height, H	1000 m
Number of array elements, N	80
Array element spacing, d	0.0006 m
Platform movement speed, v	70 m/s
Number of observations, I	30
Effective aperture, L	168 m
Equivalent bandwidth, B	266.4 MHz

**Table 2:** Evaluation of the two algorithms’ imaging performance parameters.

Imaging Algorithm	TBR/dB	ENT
BP 2D algorithm	12.9946	6.3360
Deconvolution 2D algorithm	35.8598	2.6994

## Data Availability

No new data were created or analyzed in this study.
